# Histology and Cytopathology Capacity in the Public Health Sector in Kenya

**DOI:** 10.1200/JGO.17.00122

**Published:** 2017-12-07

**Authors:** Nathan R. Brand, Nicholas Wolf, John Flanigan, Richard Njoroge, Alfred Karagu

**Affiliations:** **Nathan R. Brand,** Consultant for Leidos Biomedical, Frederick; **Nicholas Wolf** and **John Flanigan,** National Cancer Institute, Bethesda, MD; **Nathan R. Brand,** Columbia University College of Physicians and Surgeons, New York, NY; **Richard Njoroge** and **Alfred Karagu,** Ministry of Health; and **Alfred Karagu**, National Cancer Institute of Kenya, Nairobi, Kenya.

## Abstract

**Purpose:**

Histology and cytopathology services are necessary for cancer diagnosis and treatment. However, the current capacity of Kenya’s pathology laboratories is unknown. A national survey was conducted among public sector pathology laboratories to assess their capacity to perform histology, fine-needle aspiration, and bone marrow aspiration.

**Methods:**

Between April and June 2017, we identified all public hospitals that provide pathology services in Kenya. In total, two national and 13 county referral hospitals met the inclusion criteria and were sent a standardized, pretested, self-administered questionnaire.

**Results:**

A total of 11 hospitals (73%) completed the survey. The reported total caseload of histology, fine-needle aspiration, and bone marrow aspiration for 2016 was 26,472. All of the facilities staffed a pathologist and were providing cancer-related diagnostic services. Nine (82%) of the hospitals maintain a register of diagnosed cancer cases, but only one (11%) of those uses an electronic system. Six (55%) of the surveyed hospitals were able to perform histology with a median turnaround time of 14 days. Six (55%) laboratories regularly referred some specimens elsewhere for interpretation, but three of these centers relied on patients for transportation of the specimen to the referral institution. No laboratories were accredited by an external organization; however, 10 (91%) of the laboratories were working toward achieving accreditation, but only for clinical pathology services.

**Conclusion:**

This study describes the current status of histology and cytopathology capacity in Kenya’s public sector hospitals. It provides useful baseline information needed by the Ministry of Health to develop necessary capacity building and referral-strengthening interventions. A high proportion of hospitals are working to achieve accreditation points toward their commitment to providing quality services to the Kenyan public.

## BACKGROUND

Cancer is a significant cause of mortality in Kenya; it is estimated that in 2012, the number of new cancer cases was 40,999 with a mortality of 28,500.^1^ For a majority of cases, the diagnosis of cancer requires a functional pathology laboratory. However, despite its importance, there has been no previous effort to provide a detailed description of the capacity of histology and cytopathology, which, for the purposes of this study, refers to fine-needle aspiration (FNA) and bone marrow aspiration (BMA) services in Kenya. Registry data from Nairobi County show that 85% of patients treated for cancer between 2004 and 2008 had a tissue diagnosis,^2^ but this figure is probably artificially elevated as a result of the reliance of the registry on pathology laboratories as a data source, causing patients who are unable to access pathology services to be less likely to be captured in the registry. In addition, the Nairobi registry only captures data on citizens who reside in the capital county, where access to pathology services is significantly greater than in the rest of the country. Previous studies report that in Kenya, the pathologist to population ratio is 1 to > 725,000.^3^ Given this shortage of available pathology services, it is imperative that investments are made to ensure that the available resources are used as efficiently as possible so that the entire population has access to accurate and efficient cancer diagnostics.

Strengthening the capacity of pathology services is a priority of both the Kenyan Ministry of Health (KMOH) and the National Cancer Institute of Kenya (NCI-K). In the first half of 2017, KMOH and NCI-K led a multistakeholder effort to write a new national cancer control strategy that will guide the country in its cancer control efforts from 2017 to 2022.^4^ A key priority of the Cancer Diagnosis, Registration and Surveillance section of this new strategy is to complete a situational analysis of the existing capacity of pathology services in Kenya.^4^ The importance of this activity as a first step in understanding the current landscape before designing an intervention has been echoed elsewhere in the literature^5^; however, it has not been completed in any country in the region.

In Kenya, the private sector, public sector, and faith-based organizations all offer health care. In the private sector, advanced cancer diagnostic services are available. However, only 10% of Kenya’s population is covered by any form of health insurance and has access to private health care, forcing a majority of the population to rely on the public sector.^6-8^ Considering this, as well as the National Cancer Control Strategy’s focus on strengthening public sector health services, we decided that this assessment would focus on the public sector, where the KMOH and NCI-K have the greatest influence to effect change.

## METHODS

The KMOH and NCI-K, with technical support from the US National Cancer Institute’s Center for Global Health, conducted an assessment of the capacity of Kenyan public sector pathology laboratories to provide histology and cytopathology services. A survey was designed and pilot tested at Aga Khan University Hospital, a private university hospital in Nairobi. The inclusion criterion for our study was any national, county, or subcounty hospital with a pathology laboratory. Fifteen hospitals, including 13 county hospitals and the two national referral hospitals, met the inclusion criterion.

The survey was sent electronically to all 15 institutions as a Microsoft Word (Microsoft, Seattle, WA) attachment and as a link to an online version through Google Forms (https://goo.gl/forms/7wmPyeqVan4P0xFj2; Google, San Francisco, CA). For data entries that did not use the online link, N.R.B. entered data from the Microsoft Word document into Google Forms. The data were then exported to Microsoft Excel before being imported into Stata12 SE (StataCorp, College Park, TX) for analysis using simple descriptive statistical analysis.

**Fig 1:**
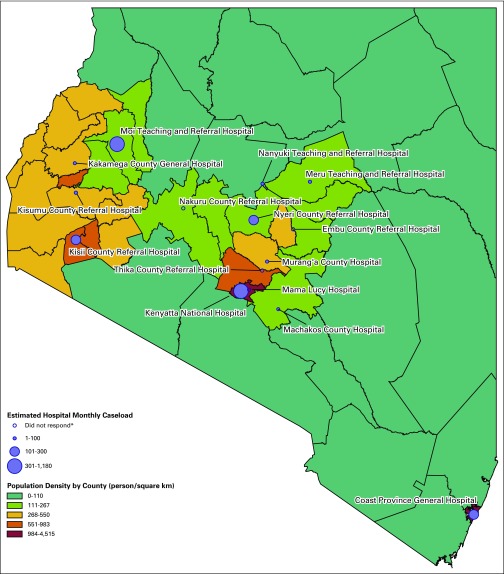
Surveyed hospital locations and population density of all 47 Kenyan counties in persons per square kilometer. Size of marker corresponds to estimated monthly caseload. (*) Embu County Referral Hospital, Nakuru County Referral Hospital, and Thika County Referral Hospital did not respond to the survey.

To create Figure 1, the population densities in persons per square kilometer from the Kenya Population and Housing Census 2009^9^ were mapped to their respective counties in ArcGIS (ESRI, Redlands, CA). The Kenya shapefile is located at https://www.arcgis.com/home/item.html?id=5f83ca29e5b849b8b05bc0b281ae27bc. We then overlaid the location of the 15 hospitals included in the study, with the size of the location marker corresponding to the monthly reported caseload of the hospital. Caseload was defined as the sum of the histology, FNA, and BMA interpretations done in an average month, as reported in the survey.

The study was granted exemption by the Office of Human Subject Research Protection at the United States National Institutes of Health because the survey did not ask for any personal identifying information or any personal opinions from those surveyed.

## RESULTS

The survey was sent to 13 county hospitals and two national referral hospitals. Responses were received from 11 hospitals, yielding a response rate of 73%. According to KMOH records, of the four nonresponders, two did not have a pathologist, and two had an active pathology program but did not respond to the survey. The locations and estimated caseload of all histology, FNA, and BMA interpretations for all disease processes in 2016 are shown on a population density map of Kenya (Fig 1). Cumulatively, there were an estimated 18,000 histology; 6,600 FNA; and 1,872 BMA interpretations during 2016 for a caseload of 26,472. Of this total, the two national referral hospitals, Moi Teaching and Referral Hospital and Kenyatta National Referral Hospital, contributed 18,780 (71%) of the entire national caseload.

Of the hospitals that responded to the survey, all 11 laboratories staffed at least one pathologist, yet six (55%) of these hospitals reported not having a pathologist for at least 1 month within the past year (Table 1). Common reasons cited were annual leave and the 100-day physician strike from December 5, 2016, to March 15, 2017. Only six (55%) of the surveyed hospitals had the capacity to perform histology, and for these the median turnaround was 14.5 days (range, 7 to 21 days). Reasons for the delay included pathologist and technologist availability and equipment downtime. All of the hospitals without histology capacity reported lack of proper equipment or supplies as the barrier to offering these services. Nine (82%) of the hospitals maintain a record of diagnosed cancer cases, but only one (11%) uses an electronic system. Of those that maintain a register of diagnosed cancer cases, only two do so with internationally standardized and evidence-based reporting protocols. Finally, although none of the laboratories surveyed were accredited by an external organization, 10 (91%) of the laboratories were working toward such accreditation, with 70% using the Stepwise Laboratory Improvement Process Towards Accreditation (SLIPTA) program.^10^

**Table 1:**
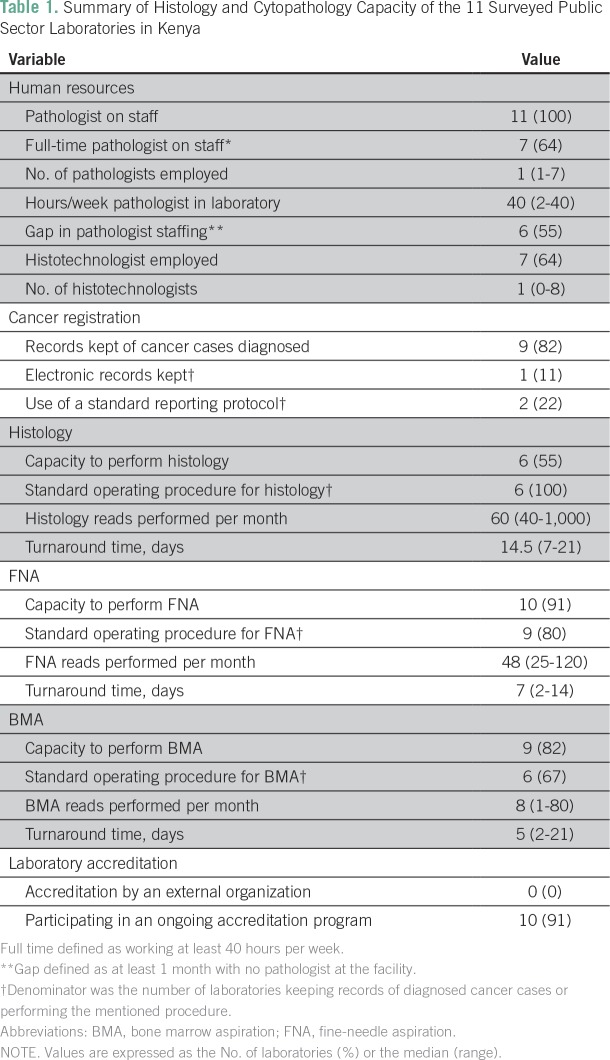
Summary of Histology and Cytopathology Capacity of the 11 Surveyed Public Sector Laboratories in Kenya

Six (55%) of the hospitals referred some specimens to another laboratory or hospital on a monthly basis (Table 2). Three of the laboratories had a formal agreement with the referral institution or laboratory, and three did not. Of the six hospitals that referred specimens on a regular basis, five partially or entirely relied on the patient to transport the specimen to the laboratory accepting the referral; only one laboratory offered a KMOH courier service to transport the specimen. All of the hospitals that did not refer specimens reported that they desired a formal referral network with other laboratories to provide second opinions and tests not offered on site.

**Table 2:**
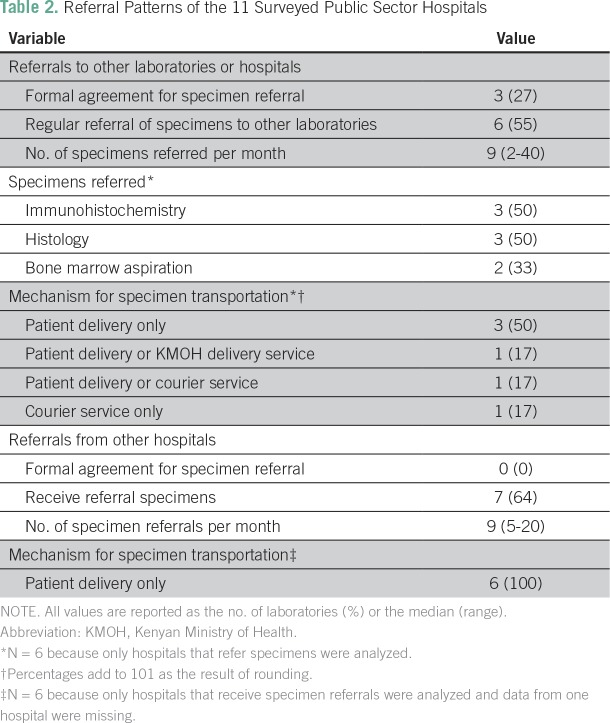
Referral Patterns of the 11 Surveyed Public Sector Hospitals

## DISCUSSION

This study describes the capacity of histology and cytopathology laboratories in the public sector in Kenya and identifies areas in need of support to strengthen Kenya’s diagnostic capacity for cancer. Overall, our results were similar to those found in an Africa-wide assessment of pathology resources for oncology care, but direct comparison between the studies is difficult because the mentioned study relied on four or fewer responders per country from the region for its data.^3^ To our knowledge, this study is the first time that a country in East Africa has completed an in-depth analysis of its histology and cytopathology services nationally. As such, it can serve as an example to other countries interested in this important first step to strengthening their national cancer diagnostic system.

Currently, Kenya has a population of 46.8 million people.^11^ It is estimated that in 2012, Kenya had an incidence of 40,999 cancer cases.^1^ However, in the public sector, this study estimates that in 2016, the total caseload of histology, FNA, and BMA interpretations was 26,472 for all disease processes, highlighting the significant deficit in the capacity of the public sector’s pathology laboratories. This means that even if each histology, FNA, and BMA interpretation led to a new cancer diagnosis, the public sector would have only diagnosed 65% of the estimated new cancer cases in Kenya. However, this is a significant overestimation because these tests are not always diagnostic and are also vital modalities for the diagnosis of nonmalignant diseases. This is highlighted by a recent study of the surgical pathology specimens (mainly from mass lesions) analyzed by Partners in Health in Haiti and Rwanda, which found that only 50% of pathology specimens led to a malignant diagnosis and that 12% were analyzed as part of work ups to diagnose infectious or inflammatory disease processes.^12^ If these data were generalized to the Kenyan context, it would suggest that the public health care system in Kenya is currently diagnosing < 35% of all new cancer diagnoses in the country.

Figure 1 illustrates that public sector pathology laboratories are generally distributed in areas of high population density. However, the figure also makes it clear that there are both large areas of low population density with no available laboratory, as well as regions in central and western Kenya with high population density where access to pathology could be improved. In addition, the figure demonstrates that the total caseload is not distributed evenly among all centers; rather, the majority of laboratories are low volume compared with Moi Teaching and Referral Hospital and Kenyatta National Hospital, the two national referral hospitals. These two hospitals see a majority of the cancer cases treated in the public sector and made up 71% of Kenya’s histology, FNA, and BMA caseload in the public sector in 2016. In Uganda, the relationship between laboratory volume and quality has been studied, and it was found that low-volume centers were significantly associated with low-quality services.^13^ This suggests that it may be more efficient to invest in a strong referral network and increase capacity at existing laboratories, especially those at the national referral hospitals, rather than invest in new small-scale centers in regions that currently lack public sector histology and cytopathology services. This would allow the expertise that already exists in the national referral laboratories to benefit smaller county hospitals, which face significant challenges in providing cancer diagnostic services to their catchment areas.

Fleming et al^14^ recently described the essential pathology laboratory package for a lower- and middle-income country and called for a tiered laboratory system connected by integrated referral networks. Applying these norms to the Kenyan context would require each of the county and national referral hospitals to have the capacity to perform histology, FNA, and BMA. However, this survey found that only 55% of the 11 laboratories that responded to the survey met this standard. In addition, none of the laboratories reported a turnaround for histology results within the suggested 5-day window.^14^ This can be improved by investing in increased histotechnologist and pathologist staffing in public sector laboratories, as our survey shows a significant deficit in human resources, with only 64% of laboratories employing a histotechnologist and 55% reporting a gap in pathologist staffing. Currently, these deficiencies in diagnostic capacity and turnaround time significantly hinder the public sector’s ability to diagnose and treat patients with cancer in addition to disrupting the country’s ability to perform cancer surveillance. However, with increased investment in laboratory staffing, these deficits can be improved.

A strong cancer registry is fundamental to a country’s ability to implement cancer control. It requires a pathology system both to provide accurate diagnoses as well as report these data in a centralized and organized fashion. Internationally standardized and evidence-based reporting protocols are essential to ensuring that all necessary information is included in each laboratory report. Yet these protocols are used at only 22% of laboratories that kept records of cancer diagnoses and 18% of laboratories surveyed. The Kenyan National Cancer Registry previously identified this as a barrier to cancer surveillance.^15^ It will be important to invest in increasing use of synoptic reporting protocols in public sector laboratories so that the registry is able to abstract complete data from all of the cancer diagnoses made in the public sector.

Integrated and strong referral networks are essential to a national laboratory system to ensure that the entire population has access to tests that may not be offered at their closest laboratory. Of the public hospitals surveyed, six regularly referred specimens to another institution, but only three of them had a formalized referral agreement with the receiving laboratory. Furthermore, the current system relies heavily on the patient to transport the pathology specimen, which is a significant barrier to timely and efficient referral. These results demonstrate the need for major investment in formalizing the referral networks within Kenya and the creation of a service to transport specimens to ensure that all laboratories function in an integrated manner. The National Public Health Laboratory is creating an oncology reference laboratory that will be able to offer specialized cancer diagnostics to the country. The creation of this center provides an important opportunity for the National Public Health Laboratory to lead the effort in strengthening the national referral network for oncology specimens.

Laboratory accreditation is necessary to ensure the provision of high-quality services. None of the surveyed laboratories had been accredited by an external organization, but 91% are working to gain accreditation. The majority of these laboratories are working through the SLIPTA program, which was developed by the WHO’s Africa Regional Office to achieve International Organization for Standardization 15189 standards.^10^ The significant participation in this program by laboratories in Kenya is encouraging and shows the country’s commitment to ensuring quality pathology services in the public sector. However, although the commitment to quality is clearly demonstrated, the current SLIPTA program only pertains to clinical pathology and does not include histology or cytology services. It is also concerning that not all laboratories have standard operating procedures for all of the tests they administer. Ensuring that all laboratories have a standard operating procedure for every diagnostic test they perform should be the first step to increasing the quality of services provided and should be prioritized. Longer-term projects should leverage the success of the SLIPTA program and work on expanding its scope to include anatomic pathology.

The strengths of this study include the high response rate of 73% for all county and national referral hospitals that have a pathology laboratory and the in-depth information gathered. This provides a baseline assessment of the landscape of the public sector’s capacity for histology and cytopathology in Kenya. The limitations of this study include the reliance on self-reported data, our focus on the public health care sector, our cross-sectional design that limited us from analyzing trends, and our limited scope of data that did not include other important cancer diagnostic modalities such as trephine biopsies. In the future, there is an opportunity for studies to expand this assessment to include the private sector and faith-based organizations, as well as to follow up with site visits to the participating and nonparticipating laboratories to better identify barriers to cancer pathology in Kenya.

In conclusion, this study describes the histology and cytopathology capacity in the public sector and provides specific suggestions on how to improve diagnostic services to the entire country. Capacity to perform basic services such as histology is lacking at 45% of the public sector laboratories that participated in this study, and the current referral network needs to be strengthened. Despite these challenges, the high participation of laboratories in ongoing efforts to achieve accreditation, albeit limited to clinical pathology, shows the commitment of the public sector to providing quality diagnostics to the Kenyan public. The KMOH and NCI-K can use this baseline assessment to guide decision making on how to improve cancer diagnostic capacity in the country.

## References

[1] Ferlay J, Soerjomataram I, Dikshit R (2015). Cancer incidence and
mortality worldwide: Sources, methods and major patterns in GLOBOCAN
2012. Int J
Cancer.

[2] Korir A, Okerosi N, Ronoh V (2015). Incidence of cancer in
Nairobi, Kenya (2004-2008). Int J
Cancer.

[3] Nelson AM, Milner DA, Rebbeck TR (2016). Oncologic care and
pathology resources in Africa: Survey and
recommendations. J Clin
Oncol.

[4] Kenya Ministry of
Health (2017). National Cancer Control
Strategy 2017–2022.

[5] Adesina A, Chumba D, Nelson AM (2013). Improvement of pathology
in sub-Saharan Africa. Lancet
Oncol.

[6] Mulupi S, Kirigia D, Chuma J (2013). Community
perceptions of health insurance and their preferred design features:
Implications for the design of universal health coverage reforms in
Kenya. BMC Health Serv
Res.

[7] Okech TC, Lelegwe SL (2015). Analysis
of universal health coverage and equity on health care in
Kenya. Glob J Health
Sci.

[8] Chuma J, Maina T, Ataguba J (2012). Does
the distribution of health care benefits in Kenya meet the principles of
universal coverage?. BMC Public
Health.

[9] Kenya National Bureau of
Statistics (2009). Population Distribution
By Sex, Number of Households, Area and Density By County and District, 2009
Population and Housing Census.

[10] WHO Regional Office for
Africa (2015). WHO Guide for the Stepwise
Laboratory Improvement Process Towards Accreditation in the African Region
(SLIPTA). Republic of Congo.

[11] (2017). The World
Factbook.

[12] Carlson JW, Lyon E, Walton D (2010). Partners in pathology: A
collaborative model to bring pathology to resource poor
settings. Am J Surg
Pathol.

[13] Amukele TK, Schroeder LF, Jackson JB (2015). Most clinical laboratory
testing in Kampala occurs in high-volume, high-quality laboratories or
low-volume, low-quality laboratories. A tale of two
cities. Am J Clin
Pathol.

[14] Fleming KA, Naidoo M, Wilson M (2017). An essential pathology
package for low- and middle-income
countries. Am J Clin
Pathol.

[15] Korir A, Gakunga R, Subramanian S (2016). Economic analysis of the
Nairobi Cancer Registry: Implications for expanding and enhancing cancer
registration in Kenya. Cancer
Epidemiol.

